# Chorea as the First and Only Manifestation of Systemic Lupus Erythematosus

**DOI:** 10.1155/2012/907402

**Published:** 2012-09-16

**Authors:** Abdul Razzakh Poil, Fahmi Yousef Khan, Abdo Lutf, Mohammed Hammoudeh

**Affiliations:** ^1^Rheumatology Division, Department of Medicine, Hamad General Hospital, Doha, Qatar; ^2^Department of Medicine, Hamad General Hospital, Doha, Qatar

## Abstract

We report a case of right-sided hemichorea associated with systemic lupus erythematosus (SLE) in a female patient who presented with involuntary movements of hand and foot, without any other manifestation of SLE. Further workup showed positive tests for antinuclear antibody, anti-Smith antibody, anti-dsDNA, and antiphospholipid antibody (aPL). The patient was started on aspirin and hydroxychloroquine and her chorea resolved after three weeks of followup. This is one of the few reported cases of SLE where chorea is presented as the first and only manifestation of SLE.

## 1. Introduction

Central nervous system (CNS) lupus is a serious but potentially treatable illness, which still presents a very difficult diagnostic challenge. The frequency of neuropsychiatric manifestations in SLE varies widely, depending on the type of manifestations and the method used for evaluation [1]. However, neurologic and psychiatric symptoms are reported to occur in 14 to 80 percent of patients either prior to the diagnosis of SLE, or during the course of their illness [2]. Chorea is a relatively uncommon manifestation of SLE; however, chorea as the first and sole manifestation of SLE is extremely rare [3]. In this paper, we present a young woman who presented with hemichorea as a first and sole manifestation of SLE. 

## 2. Case Report

A 27-year-old female presented to emergency room with involuntary movements of her right arm and leg. These movements had started 4 weeks earlier and gradually became worse, involving the right side of the body; she had difficulty in holding things with her right hand and difficulty in walking. There was no history of rash, photosensitivity, hair loss, oral ulcer, Raynaud's phenomenon, dryness of mouth or eyes, oral contraceptive intake, weight loss, headache, loss of consciousness, or seizure. She had no family history of rheumatic or neurological diseases and her past medical history was unremarkable. She denied smoking and alcohol consumption. The patient was multigravida, she had two children; both pregnancies were uneventful.

Physical examination revealed choreic movements of her right hand and foot. They were jerky, purposeless, intermittent, and irregular movements. Examination of other systems was unremarkable. 

**Figure 1:**
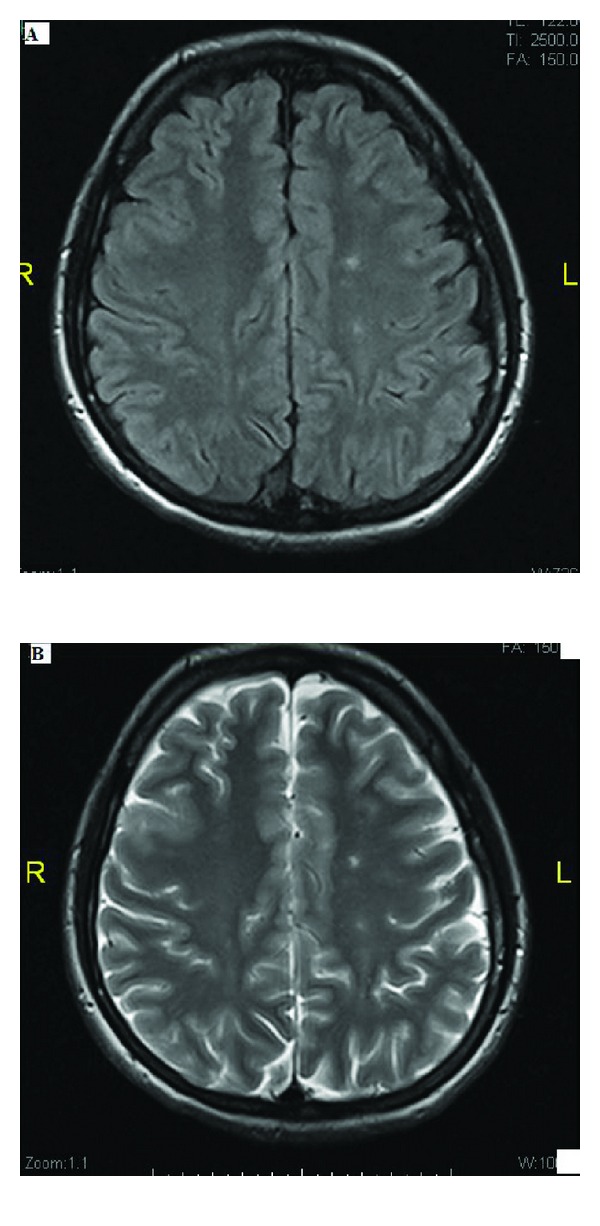
Brain magnetic resonance image (MRI) shows left periventricular white matter hyperintensities in FLAIR image (A), and left periventricular white matter hyperintensities in axial T2-weighted brain MRI (B).

Initial investigations showed a normal complete blood count, blood chemistry, and liver function tests. Her coagulation profile was normal except for prolonged activated partial thromboplastin time (APTT) of 68.8 seconds (25 sec to 36.5 second). Antinuclear antibody (ANA) was 1 : 1280, and Anti-dsDNA was 70.5 IU (<25 IU) and anti-Smith antibody was also positive. Her C3 was low and C4 complement was normal. Lupus anticoagulant was positive and anticardiolipin IgG was borderline positive-18.5 GPL (<15 GPL), but anticardiolipin IgM antibody and anti-beta 2 glycoprotein-1 were negative. Antistreptolysin O (ASO) titre was 157 IU/mL (<200 IU/mL) and thyroid function tests were normal. Magnetic resonance imaging (MRI) of brain showed tiny foci of high-intensity signal in FLAIR and T2-weighted image in bilateral basal ganglia and occipital periventricular white matter (Figures 1(a) and 1(b)). Magnetic resonance angiography (MRA) showed normal cerebral arterial caliber with no area of stenosis or occlusion, or aneurysmal dilatation. 

In view of these findings, the patient was diagnosed with systemic lupus erythematosus (SLE) and was treated with aspirin and hydroxychloroquine. After three weeks of followup, her chorea resolved completely. She was followed in rheumatology for the past 6 months without any recurrence.

## 3. Discussion

The incidence of chorea in SLE varies in different studies. It ranges from 1% to 8% [4, 5], and it is strongly associated with antiphospholipid (aPL) antibodies, especially anticardiolipin and lupus anticoagulant [6]. Although chorea usually occurs during the course of SLE, it may also be, the presenting feature of the illness, sometimes preceding other symptoms by several years [7], or it might be the sole manifestation of SLE as in our case.

There are many explanations regarding pathophysiology of chorea in SLE, one such explanation is that it is immune-mediated mechanism secondary to aPL antibodies mainly anticardiolipin IgG as in our case [6, 8]. 

Another potential pathogenic mechanism for lupus chorea is ischemia affecting the basal ganglia or the tracts connecting the basal ganglia, thalamus, and cerebral cortex. Some of these ischemic events might be related to thrombosis mediated by IgG anticardiolipin antibodies as in our patient (see MRI picture) [8, 9].

The differential diagnosis of chorea includes cerebrovascular accidents, drug intoxication, hyperthyroidism, Huntington's disease, Sydenham's chorea, and collagen vascular diseases like SLE.

In our patient, cerebrovascular accident was excluded as MRI was unremarkable. Regarding the drug history, our patient denied receiving any triggering drugs prior to this episode. Huntington's chorea was excluded from the history since there was no personal or family history of abnormal movements. Hyperthyroidism was excluded by normal thyroid function test, whereas the absence of history of rheumatic fever and antecedent streptococcal infections made the diagnosis of rheumatic fever (Sydenham's chorea) unlikely. 

Our patient does not meet the ACR criteria for SLE, but positive anti-Smith and anti-dsDNA antibodies make SLE the most likely diagnosis, since anti-Smith is exclusively found in SLE if anti-dsDNA is also present [10, 11].

One of the commonest MRI findings of neuropsychiatric SLE is periventricular white matter hyperintensities in T2 and FLAIR images, as in our case (see MRI picture) [12].

Most of the patients with chorea secondary to SLE improve without any treatment and there is no role for anticoagulation in the treatment of chorea in the absence of a thromboembolic event. Alternatively, prednisone, dopamine antagonists, and antiplatelet agents appear to be effective to treat chorea in SLE patients with antiphospholipid antibodies [8, 13]. Considering her mild chorea, we did not start her on immunosuppressive or dopamine antagonists. 

In conclusion, chorea is a well-recognized but rare manifestation of SLE; it could be the initial and the sole presentation of SLE and the diagnosis should be pursued when other causes of chorea are excluded.
